# Supplementary material for: High Red–Blue Light Ratio Promotes Accelerated In Vitro Flowering and Seed-Set Development in Amaranthus hypochondriacus Under a Long-Day Photoperiod
Source: Plants (Basel). 2025 Oct 11;14(20):3134. doi: 10.3390/plants14203134 (PMC12566660; doi:10.3390/plants14203134)
Supplement: Supplementary file 1 [file plants-14-03134-s001.zip › Figure S2.pdf]

a

b

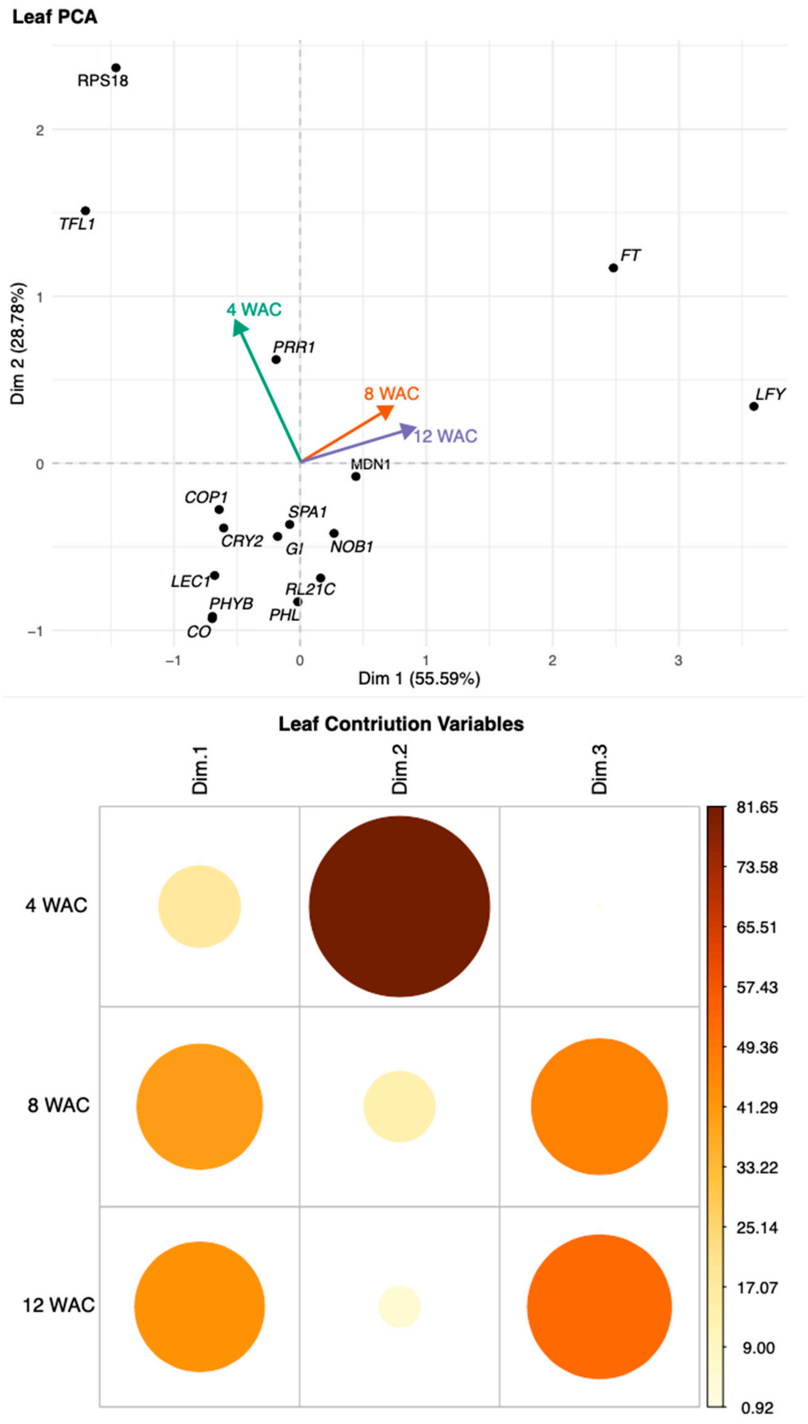


**Figure S2. Principal component analysis (PCA) of the quantitative gene expression assays detected in the leaves.** In **(a)** the PCA1 (x-axis, increasing average expression), indicates that *ca.* 56% of the genes analyzed had significantly modified levels of expression. Here, the FT and LFY genes exhibited the higher gene contribution in gene expression contribution. PCA2, (y-axis, increasing positive trend), explained *ca.* 29% of the variance It indicated that most relevant contribution to this component in leaves were *TFL1, PRR!* and *RPL18*. **(b)** Shows that components/dimensions 1 and 3 were enriched in genes whose maximum levels were mostly reached at 8 and 12 WAC, while component/dimension 2 grouped those genes whose expression was predominant at 4 WAC, representing the pre-flowering vegetative stage.
