# Supplementary material for: High Red–Blue Light Ratio Promotes Accelerated In Vitro Flowering and Seed-Set Development in Amaranthus hypochondriacus Under a Long-Day Photoperiod
Source: Plants (Basel). 2025 Oct 11;14(20):3134. doi: 10.3390/plants14203134 (PMC12566660; doi:10.3390/plants14203134)
Supplement: Supplementary file 1 [file plants-14-03134-s001.zip › Figure S3.pdf]

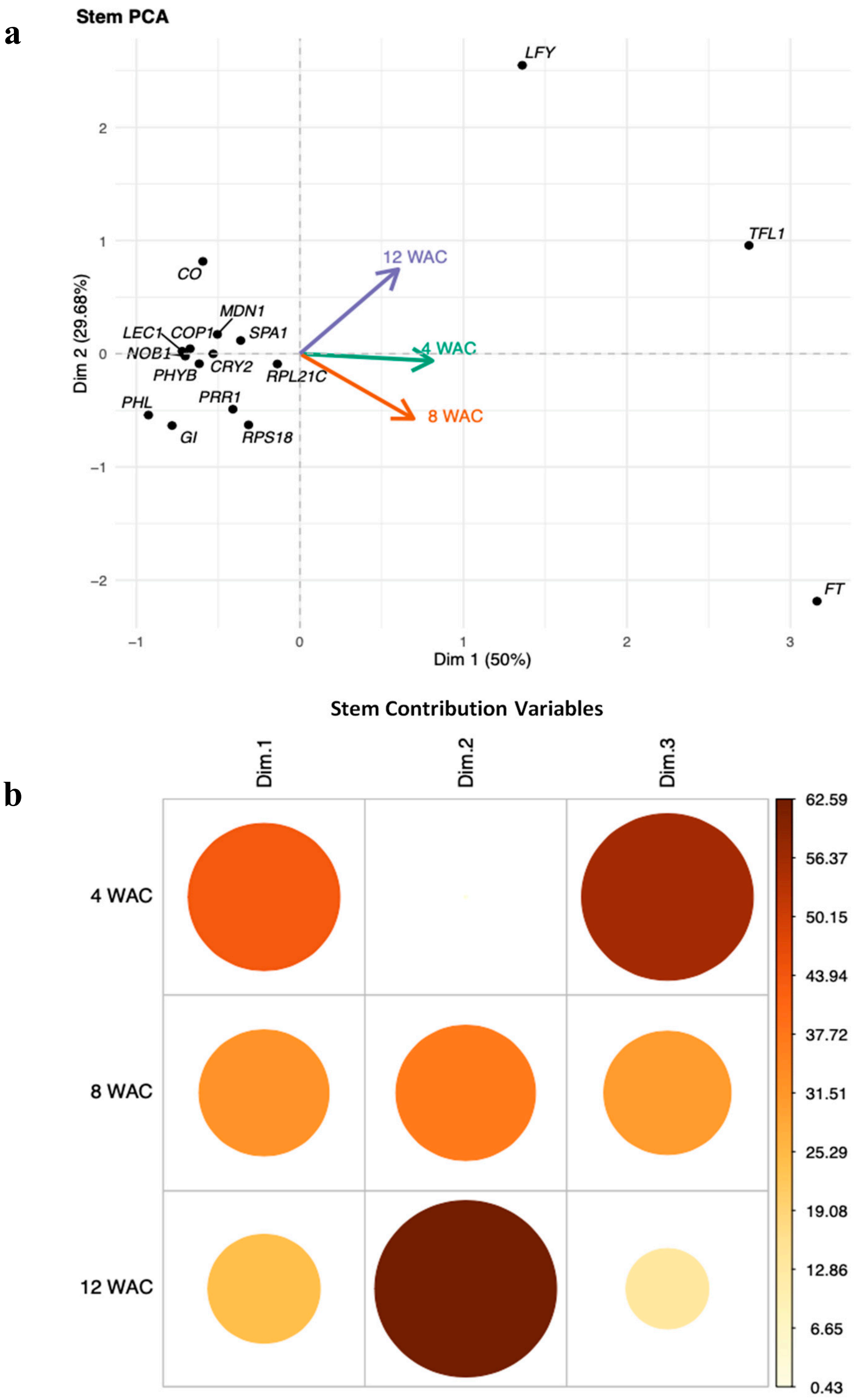

Figure S3. Principal component analysis (PCA) of the quantitative gene expression assays detected in the stems. In **(a)** the PCA1 (x-axis, increasing average expression), indicates that 50% of the genes analyzed had significantly modified levels of expression. Here, the *FT* gene exhibited the higher contribution in gene expression. PCA2, (y-axis, increasing positive trend), explained ca 29% of the variance It indicated that most relevant contribution to this component were *TFL1* and *LFY*. **(b)** Shows that components/ dimensions 1 and 3 were enriched in genes whose maximum levels were mostly reached at 4 and 8 WAC, while component/dimension 2 grouped those genes whose expression was predominant at 8 and 12 WAC.
