# Supplementary material for: High Red–Blue Light Ratio Promotes Accelerated In Vitro Flowering and Seed-Set Development in Amaranthus hypochondriacus Under a Long-Day Photoperiod
Source: Plants (Basel). 2025 Oct 11;14(20):3134. doi: 10.3390/plants14203134 (PMC12566660; doi:10.3390/plants14203134)
Supplement: Supplementary file 1 [file plants-14-03134-s001.zip › Table S1.pdf]

Table S1. **Quality of light emitted by the red/blue and white LED lamps employed in this study.** The photosynthetic photon flux (PPF) and the irrandiance were measured using a MK350S Premium Spectrometer (UPRtek, Taiwan) and a LI-COR Model LI-189 light meter (LI-COR, Lincoln, NE, USA), respectively.

	**White LED**	**Red-Blue LED**
Irradiance	200 µmol $m^{-2}s^{-1}$	94 µmol $m^{-2}s^{-1}$
PPF[1]	108.16	161.92
PPF-UV[2]	0.15	0.17
PPF-B[2]	29.30	36.10
PPF-G[2]	54.39	1.53
PPF-R[2]	24.47	124.29
PPF-NIR[2]	2.80	2.54

[1]PPF = Photosynthetic Photon Flux, in µmol s-1
[2]UV= ultraviolet; B = blue; G = green; R = red, and NIR = near infrared segments of the visible light spectrum
