# Supplementary material for: High Red–Blue Light Ratio Promotes Accelerated In Vitro Flowering and Seed-Set Development in Amaranthus hypochondriacus Under a Long-Day Photoperiod
Source: Plants (Basel). 2025 Oct 11;14(20):3134. doi: 10.3390/plants14203134 (PMC12566660; doi:10.3390/plants14203134)
Supplement: Supplementary file 1 [file plants-14-03134-s001.zip › Table S2.pdf]

Table S2: Primer design of the *A. hypochondriacus* flowering-related genes that were analyzed in the present study.

Symbol	Gene description		5' - 3' sequence
PHYB	*PHYTOCHROME B*	D[1]:<br>R[1].	ACCATGAAGCTAGACAAGGATGAG<br>GATCCCCGCAAATCGTCAGA
PHL	*PHYTOCHROME-DEPENDENT LATE-FLOWERING*	D:<br>R.	TCAGATCCGCCTTGCTAATG<br>GCACGGCGATAGTCCTTATT
CRY2	*CRYPTOCHROME 2*	D:<br>R.	TGAATTTGTTGAGCAACACCTCG<br>TTCCCCGAAATGAAGATAAGGGG
COP1	*CONSTITUTIVE PHOTOMORPHOGENIC 1*	D:<br>R.	CGCGCAAGGGATAGGTATTCT<br>GAAAACTTCCCGCGGTCAAC
SPA1	*SUPPRESSOR OF PHYA-1*	D:<br>R.	GCGACCCTTGTTTCTGCATC<br>GGCAACGGACAAACCAACAA
CO	*CONSTANS*	D:<br>R.	CCTTGGATGTGGGTGTTGTA<br>GCGATAGTTGAGTCGGTACTTG
FT	*FLOWERING LOCUS T*	D:<br>R.	CTCATCTCGAAACCTCGTGTT<br>GATGTCGGTCACCATCCAAT
TFL1	*TERMINAL FLOWER 1*	D:<br>R.	CCAGTGATCCTTACCTGAGAGA<br>CTATGTTTGGCCTTGGCATTTC
LFY	*LEAFY*	D:<br>R.	ATCCAACGCCCTAAGGAAAG<br>GCGCGGATGAGAATTAAAGATG
GI	*GIGANTEA*	D:<br>R.	AGACTGTTGCTTGGACTTCTC<br>GCATCCAGTTTCTTGGCAATC
TOC1/PRR1	TIMING OF CHLOROPHYLL A/B BINDING PROTEIN/PSEUDO RESPONSE REGULATOR1	D:<br>R.	GCAATGGCCAAGCAGTTATT<br>TCCTCACCATGCGAGTTATTT
RPS18	*CHLOROPLAST-ENCODED RIBOSOMAL PROTEIN S18*	D:<br>R.	CTTTTCGTAGGCGTTTGCCC<br>AGGTCAATCTATTCACTCGCCT

RPL21C	*RIBSOMAL PROTEIN L21 SUBUNIT C*	D:<br>R.	AGCACTTACATCGGGAAGCC<br>AGTGATGGGCTGTCTGTGAC
NOB1	*NIN1 (ONE) BINDING PROTEIN 1*	D:<br>R.	AACGTAACTGCTGAGGTGGG<br>TGTAGCACAACCCCGTTCTC
MDN1	*MIDASIN HOMOLOGUE 1*	D:<br>R.	CACTGTATGCCGTGCCCTAT<br>CCAGCTTCTCCAGTCAAGGG
LEC1	*LEAFY COTYLEDON 1*	D:<br>R.	CTTCTATGGGCGATGACTACAC<br>TATTCCCTTGAACCGGCATAC
ACT	*ACTIN*	D:<br>R.	CGTGACCTGACTGATTACCTTA<br>GCTCGTAGTTCTTCTCAATGGC
TUB	*TUBULIN*	D:<br>R.	TCTCAGCAGTATGTCTCCCTCA<br>TCTACTTCTTTGGTGCTCATCTT
EF1-α	*ELONGATION FACTOR 1-ALPHA*	D:<br>R.	GCCAAATATCTAAGAAACAAATGC<br>TAGCACAACCACATGATATTTCTT

[1]D = Direct; R = Reverse
