# Supplementary figures and images for: High Red–Blue Light Ratio Promotes Accelerated In Vitro Flowering and Seed-Set Development in Amaranthus hypochondriacus Under a Long-Day Photoperiod
Source: Plants (Basel). 2025 Oct 11;14(20):3134. doi: 10.3390/plants14203134 (PMC12566660; doi:10.3390/plants14203134)

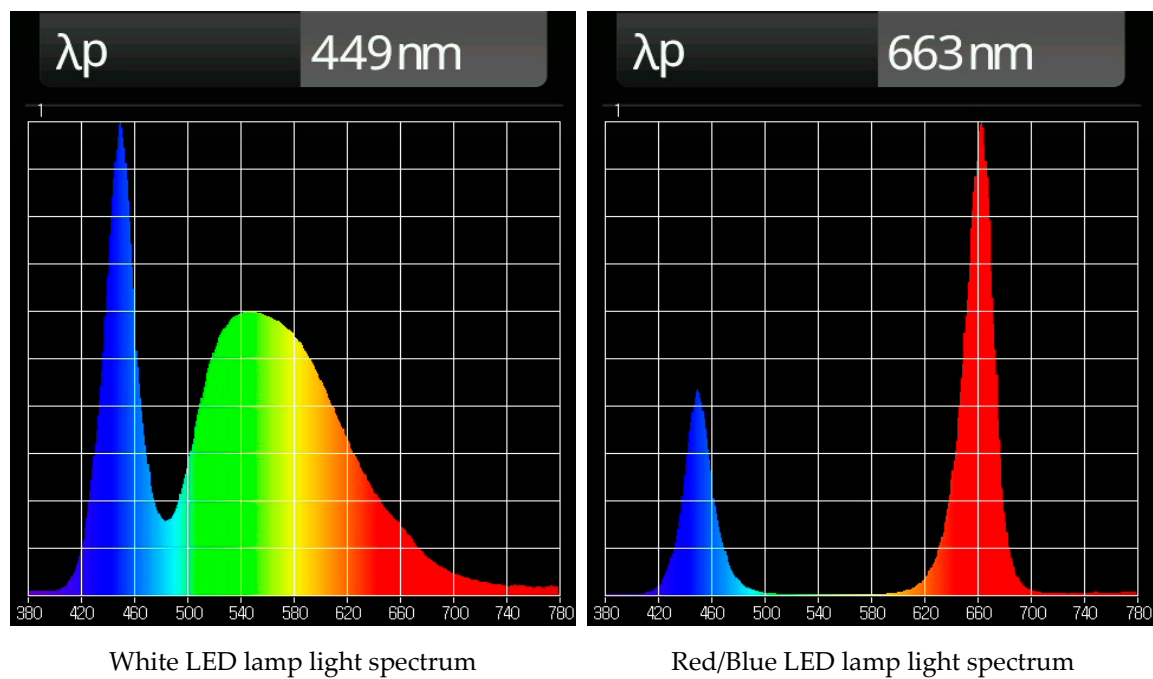

**Figure S1.** Light spectra of the white and red/blue LED lamps employed in this study.

Supplement: Supplementary file 1 [file plants-14-03134-s001.zip › Figure S1.pdf]
